# An organisational analysis of the implementation of telecare and telehealth: the whole systems demonstrator

**DOI:** 10.1186/1472-6963-12-403

**Published:** 2012-11-15

**Authors:** Jane Hendy, Theopisti Chrysanthaki, James Barlow, Martin Knapp, Anne Rogers, Caroline Sanders, Peter Bower, Robert Bowen, Ray Fitzpatrick, Martin Bardsley, Stanton Newman

**Affiliations:** 1 University of Surrey, Department of Health Care Management and Policy, University of Surrey, Guildford, UK; 2 Imperial College Business School, London, UK; 3 PSSRU, London School of Economics and Political Science, London, UK; 4 University of SouthamptonHealth Sciences, Southampton, UK; 5 Institute of Population Health, University of Manchester, Manchester, UK; 6 University of Oxford, Public Health, Oxford, UK; 7 The Nuffield Trust, London, UK; 8 City University London, Health Services Research; UCL, Cardiovascular Sciences, London, UK

**Keywords:** Telecare, Telehealth, Whole system redesign, Organisational change, Adoption, Implementation, Ethnographic methods

## Abstract

**Background:**

To investigate organisational factors influencing the implementation challenges of redesigning services for people with long term conditions in three locations in England, using remote care (telehealth and telecare).

**Methods:**

Case-studies of three sites forming the UK Department of Health’s Whole Systems Demonstrator (WSD) Programme. Qualitative research techniques were used to obtain data from various sources, including semi-structured interviews, observation of meetings over the course programme and prior to its launch, and document review. Participants were managers and practitioners involved in the implementation of remote care services.

**Results:**

The implementation of remote care was nested within a large pragmatic cluster randomised controlled trial (RCT), which formed a core element of the WSD programme. To produce robust benefits evidence, many aspect of the trial design could not be easily adapted to local circumstances. While remote care was successfully rolled-out, wider implementation lessons and levels of organisational learning across the sites were hindered by the requirements of the RCT.

**Conclusions:**

The implementation of a complex innovation such as remote care requires it to organically evolve, be responsive and adaptable to the local health and social care system, driven by support from front-line staff and management. This need for evolution was not always aligned with the imperative to gather robust benefits evidence. This tension needs to be resolved if government ambitions for the evidence-based scaling-up of remote care are to be realised.

## Background

The need for new models of integrated care that can reduce the costs of keeping people out of care homes and hospitals is an imperative for heath and social care systems around the world. The use of ‘remote care’ technologies (telecare and telehealth) as integral to new models of care is seen by the UK government as one potential solution [1]. Since the mid 2000s, a number of programmes have been launched in the UK to stimulate the adoption of remote care, including in the Department of Health’s Whole System Demonstrator (WSD) programme. The WSD had two main goals. The demonstration aspect refers to delivering a model that results in more integrated working practices across the NHS (health care) and Local Authorities (social care) at organisational and routine service delivery levels, supplanting traditional models of care through increased use of telehealth and telecare services. The second goal was to test the wide scale impact of *telehealth* (the remote exchange of data between a patient, at home, and health care professionals, to assist in the management of an existing long-term condition i.e. COPD, diabetes, heart failure), and *telecare*, (the remote, automatic monitoring of an individuals’ personal health and safety, i.e. mobility, and home environment). At the start of the WSD programme, although there was some evidence for the clinical and cost effectiveness of telehealth and telecare [2-4], the evidence base was not so rigorous that wide-scale national adoption has been achieved [5,6]. Combined implementation and evaluation of remote care is complex, with the ease of the task and the success achieved often overestimated [5,7]. Despite the Government promoting the concept [6,8], many seemingly successful telehealth and telecare projects fade away [9,10].

The idea of using remote care as a driver of whole-system working and integration has been seen politically as attractive [1], with this concept largely treated as ‘self-evident and readily available for operational use’ [11]. In reality the term a ‘whole system’ approach is used ubiquitously and tends to be a non-specific policy goal, with what it means, and how it could be achieved left ambiguous [11,12]. In this instance it has been used to denote telecare as part of a joined-up health and social care system. By commissioning this work the UK Department of Health wanted to move away from remote care merely as a ‘good idea’, by providing future policy makers with formal and definitive ‘proof’ that this technology worked [8], underpinned by a normative expectation that only the outcomes of a large and robust randomised controlled trial (RCT) would do. The overall WSD evaluation was therefore nested within, reportedly, the world’s largest RCT of remote care technologies to date [13]. Findings from WSD are now becoming available as they go through peer review [14,15]. The papers focus on (1) clinical outcomes and patient reported outcomes, (2) changes in service use and economic impacts, (3) service users and carers experiences, and (4) the organisational, supply chain, and service delivery implications for scaling-up. Whilst an RCT may provide a suitable vehicle for demonstrating the impact of clinical and cost effectiveness, determining the ‘success’ of policy concepts such as ‘whole system’ change is more challenging [8,11,12]. Currently, we have varying degrees of knowledge about the impact of innovative technologies on different aspects of the health and social care system [7,9] but require knowledge about how these elements recursively interact to achieve whole system integration and change [16]. To investigate this question we drew on a body of work that explores the task of evaluating services making the transition from clinical trials to more mainstream normalized services [5,6,8,12,17], and also embedded our approach within a wider body of literature on socio-technical change in complex organisational settings [18-20]. This body of work emphasises a constructionist ontology and interpretative epistemology in which the constituent parts of a system cannot be understood objectively, or in isolation, but need to be situated in dynamic relation to how different stakeholders ‘make-sense’ of the context and system around them [7,8,20,21].

### Research objectives

We were tasked with conducting an organisational evaluation, in parallel with service delivery implementation and an RCT. Our aim was to better understand the interconnections that exist between policy, organisational environments, contextual influences (e.g. history of remote care involvement at each site) and the programme intervention itself. In particular, we were interested in understanding the interaction of organisational factors that would assist in the successful large scale implementation of remote care. Essentially, we explored the context, mechanisms, and outcome relationships between different stakeholders across the system, by asking ‘what makes sense and works, for whom, and under what conditions’ [21]. A fundamental challenge for the sites was the anticipated tensions between differing organisational priorities: (1) to maintain an effective intervention at a high level of quality and create robust scientific evidence about the technologies in use and (2) to establish practices and systems in place that could foster sustainable use of the technologies in the future; nationwide, beyond the WSD programme.

## Methods

We conducted an in-depth, comparative, longitudinal analysis of the implementation of telehealth and telecare within the three sites, focusing both on processes and outcomes. The case study method is particularly useful where the range of issues is wide, the concepts are related to each other in complex ways [22] and context is important [23].

### Case study sites

Each WSD site was chosen by the Department of Health in 2006 through a competitive process because (a) they were considered the most likely to succeed in scaling up remote care as part of a whole system redesign and (b) they were considered representative of the range of local health and social care systems in the UK. Each site is a region of England (Cornwall, Kent, London Borough of Newham) comprising one or two (Kent) health authorities (i.e. Primary Care Trusts) and geographically superimposed Local Authorities, with responsibility for social care. Each of the three sites has its own particular characteristics and population health needs and demands. Newham has a multi-ethnic community with high levels of economic, social and educational deprivation. Kent comprises a mixed urban and rural environment, with varying levels of economic and social development. Cornwall is predominantly rural, a large, sparsely populated area with a large elderly population and poor transport links. The sites began work in 2008, to develop care models based around the introduction of new telecare and telehealth services. Implementation was nested within a large pragmatic cluster-randomised controlled trial (RCT). Pragmatic RCTs are more flexible to adjust to real life conditions, and useful when the both the context and the intervention itself (telecare and telehealth) are complex [24]. The trial was developed with two parallel treatment arms to assess the effectiveness of telehealth and telecare on healthcare utilisation/ costs and cost-effectiveness relative to standard care over a 12-month period [13]. All study sites had exactly the same type of patients enrolled in the trial, and received the same interventions. This was a stringent condition of the protocol. Each site had a local WSD Project Team with responsibility for implementing the trials in their region in line with the protocols provided by the WSD Evaluation Team. The WSD Project Teams’ responsibilities included: identifying and recruiting eligible participants; installing and maintaining telehealth or telecare devices for intervention participants; training participants (and professionals) in the use of the telehealth and telecare; providing monitoring centre services; providing usual health and social care to all participants; providing local organisational and management resources as required to support the trial. The WSD Project Teams comprised of existing staff from the local Primary Care Trust (Cornwall), Social Services (Kent) or a combination of both (Newham). All WSD Project Teams were supported by frontline clinical and technical staff. Implementation was supported across local sites by involvement of the voluntary sector, the private sector and expert patient and carer groups, who advised on the types of technology employed, helped to source patients, and advised on patient information needs. An external management consultancy appointed by the Department of Health provided additional programme support. For a detailed outline of the trial methods see Bower et al [13]. By attending early evaluation team meetings, we observed that taking part in the trial was perceived by the organisations as an exciting opportunity, that the trial would provide them with the financial and management support (Department of Health funding and a team of specialist management consultants) they needed to deploy telehealth, in particular, on a large scale. Bidding to be part of the WSD programme had been rigorous, and the site teams felt privileged to be given the opportunity to develop new services that would not otherwise have been afforded. However, once the trial got underway excitement was tempered by the level of work involved in developing new services and using technology standardised within the context of an RCT, and by the effort of ensuring that large numbers of trial participants were recruited within a relatively short timeframe (see below).

### Data collected

Qualitative, ethnographic data was collected across the sites between June 2008 and July 2011 (see Table 1). Each site had a dedicated management team charged with rolling out the programme, recruiting staff and reviewing progress. They also facilitated and co-ordinated installation of the technology, and oversaw recruitment, assessment and monitoring of patients in the trial. We purposively sampled and interviewed everyone in each site within their management teams, as well as other staff strategically involved in implementation. No-one we approached refused to participate in our study. Saturation of the findings was reached approximately half way through each of our three phases of data collection (each data collection phase represented a different stage of the programme), with the same themes re-emerging across different groups. The emphasis in the interviews was on key decisions taken by local health and social care stakeholders involved in the WSD programme, and their impact on implementation outcomes. Interviews covered the role of strategic policy, operational decisions and targets, and relationships between the organisations and different professional groups within the care system. We explored the impact of the WSD programme on local implementation practices, and on closing the traditional cultural and practice gap between health and social care. Over the trial period we conducted 115 interviews, triangulated with 92 strategic documents and 174 hours of ethnographic observations (see Table 1). Before data collection began the study was approved by Liverpool Research Ethics Committee (ref: 08/H1005/4). The data presented is part of much larger body of work currently being written up by the authors.

**Table 1:** Data collection June 2008 - July 2011

**Study timetable**	**Interviews conducted**	**Observations (on site and strategic meetings)**	**Document review**
**Pre-trial phase:** Planning and early implementation July – Oct. 2008	5 LA managers	11 x 3 hr local site management meetings	14 meeting notes
	1 NHS manager	2 x 3 hr strategic WSD team board meetings	1 WSD evaluation proposal
	8 other associated staff		1 ministerial document
		1 x 4 hr national remote care expert network meeting	3 presentations regarding remote care progress and initiatives in the UK
**Total:**	**14**	**43 hours**	**19 documents**
**Phase I:** Participant recruitment and clinical engagement	14 LA managers	10 x 3 hr local site management meetings	17 meeting notes
	14 NHS managers	5 x 3 hr strategic WSD team board meetings	3 site project management documents, 1 Newham telecare care model overview, 1 Newham telecare procedures and 1 Newham telehealth response interim care pathway documents
Nov. 2008 – Mar. 2008	3 joint LA & NHS managers	4 x 4 hr national remote care expert network meetings	
			12 presentations about remote care progress and initiatives in the UK
**Total:**	**31**	**57 hours**	**35 documents**
**Phase II:** Delayed delivery group joins the trial and the focus shifts to evaluation Sept. 2009 - Oct. 2010	14 LA managers	10 x 3 hr local site management meetings	20 meeting notes
	14 NHS managers	3 x 3 hr strategic WSD board meetings	1 telehealth pilot report
			15 presentations about remote care progress and initiatives in the UK
	1 joint LA & NHS manager	5 x 4 hr national remote care expert network meetings, 1 x 6 hr conference and 1 x 3hr launch of Kent telehealth pilot event	
**Total:**	**29**	**68 hours**	**36 documents**
**Phase III:** Business continuity plans and early mainstreaming days April – July 2011	10 LA managers	2 x 3hr local site management meetings	2 meeting notes
	14 NHS managers		
	17 other associated staff		
**Total:**	**41**	**6 hours**	**2 documents**
**Overall total:**	**115 interviews**	**174 hours of observations**	**92 documents**

### Data analysis

Our organisational analysis drew on recent innovation research [18,20,25] and normalisation process theory [19]. In paying attention and situating the findings within the dynamic complexity of the system, we mainly drew on the work of Greenhalgh and colleagues [20,21] and earlier work on the implementation of remote care and healthcare ICT [26-28]. When examining the socially constructed meanings of different stakeholders, we found the sensemaking work of Weick useful [29]. Data was analysed in three stages. Our analytic process drew on the structured and systematic approach of coding and theme abstraction [30]. To ensure reliability, two members of our team independently read interview transcripts to agree on the emerging themes. The reliability of our interpretation of the data was further established by drawing on additional data collected from the informal interviews, through observations and from project documentation (see Table 2). 

**Table 2:** Study site characteristics

**Sites**	**Cornwall**	**Kent**	**Newham**	**Overall Total**
**WSD Organisational lead**	Cornwall Primary Care Trust	Kent County Council	London Borough of Newham	
**Telecare users pre-trial**	<1000	c.2000	c.2500	5500
**Telehealth users pre-trial**	0	c.200	0	200
**Trial telecare users**				
Intervention group	545	427	304	1276
Control group	492	462	370	1324
**Total**	**1037**	**889**	**674**	**2600**
**Trial telehealth users**				
Intervention group	566	583	456	1605
Control group	625	595	405	1625
**Total**	**1191**	**1178**	**861**	**3230**
**Overall numbers (telehealth/telecare)**	**2228**	**2067**	**1535**	**5830**

## Results

By the end of the WSD programme telehealth and telecare had been rolled out across all three sites, with 2,281 participants in the intervention group and 2,949 in the control group (see Table 2).

### Whole system redesign

In the original WSD research protocol, three types of participants were to be recruited: those assessed as eligible for telecare, those assessed for telehealth, and a mixed group assessed as eligible for both [13]. However, early on in the trial it became clear that there were not the numbers expected, hence recruitment of participants with mixed care needs proved difficult. Some site managers attributed this to population demographics, but the majority of the staff believed that this group failed to be identified and recruited due to the strict RCT recruitment conditions. Whatever the reason, a lack of recruitment meant this group was not included as a separate category in the trial. This decision had far-reaching strategic consequences for the sites and their overall goal of demonstrating the advantages of whole system redesign and of providing implementation lessons for scaling-up. Health care and social care staff no longer needed to work together to deliver care for this mixed group. Subsequently, telecare and telehealth no longer acted as a combined driver for the redesign of care services, and for developing new levels of integration, because the two services remained as before, largely independent, with a range of associated but separate processes.

"I would describe it actually not as being a whole system…because of the segregation between telehealth and telecare. I think we’ve called it whole system and it’s not. I think it is two separate systems that have the potential of being the whole but… as part of the trial there was no group which had both telehealth and telecare equipment installed… It wasn’t a proper evaluation strand…it should have been (senior manager)."

In addition, new systems for data sharing across health and social care sectors failed to be developed, and the need for new organisational processes and staff that seamlessly spanned both sectors was also eliminated. Alongside any strategic vision of integrated processes, the organisational will to develop an integrated model around new combined telecare and telehealth services was dissipated. The lack of integration was not only due to RCT selection criteria. There appeared to be a lack of organisational readiness. Here readiness is defined as the degree to which the cases involved were prepared to participate and succeed in this endeavour [31,32]. We also considered the extent to which the organisations and individuals within it perceived the change as needed [32,33]. As one clinician put it; ‘*As an organisation, are they ready to change completely the way they work? And are there clinicians ready for that…I think we have proved often that they aren’t.’* To take part in the programme, sites hadto demonstrate “a history of successful partnership working across health and social care, e.g. joint health and social care teams which provide comprehensive and integrated packages”, and show “evidence of a clear plan for a whole system approach” [1]. However, the development of ‘whole system’ strategies and activities that should have commenced at the start of the programme was never pursued. Managers did not elucidate nuanced understandings of ‘whole system’ working or redesign. Overall, they were unconcerned about the specific goal of increased integration, which was seen as largely unrealistic and secondary to the development of expanded remote care services, seen as essential for improving patient care. Despite, the pre-trial conditions, we found that whole system working was not a large part of the culture being enacted and driven by staff. In practice, there was little evidence of integrated services, or any move towards integration, with services largely operating within traditional cultural, structural and financial silos and sector boundaries.

"A person in the NHS cannot create a care package in social services even though they’ve been talking about how to do it for the last five years and I can see why, because the NHS doesn’t want… well the council doesn’t want the NHS spending its budget. While you can talk about philosophy as much as you like, until there’s an integrated budget system, it’s never going to work (healthcare professional)."

The WSD programme may not have led to the ‘whole system’ change, in terms of ‘*truly integrated services’* and a ‘*radical and sustained shift in the way in which services are delivered’*, but engagement in the programme, nevertheless, strengthened existing links between health and social care. Improved communication due to the work of rolling out telehealth services in particular, acted as a catalyst for building relationships and for the joint ownership of new care processes and goals. For example, specialist community nurses responsible for telehealth monitoring worked closely with social care staff to ensure the new services fitted within patient’s existing care packages and that all members of the care team were kept informed and updated. The act of ‘*bringing people together’* was seen as a significant challenge and an important outcome in its own right, a rare ‘*gem*’ afforded by the dedicated work and funding attached to the programme. Interviewees felt that implementing the WSD programme helped to identify duplications in the existing services provided, and enabled them to work more closely around the needs of individual patients. However, many of these positive changes appear attached to the particular hard work of trying to get telehealth implemented, within the WSD trial.

### Implementation challenges in the context of an RCT

The pragmatic design of the RCT allowed for a degree of flexibility, with local sites able to choose some criteria for patient inclusion. Despite this flexibility, standardised elements of trial, such as participant recruitment processes, were perceived as problematic to local implementation processes. Local managers felt that the RCT evaluation required a ‘one size fits all’ approach that overrode local contextual differences in terms of population needs and prior experiences. For example, the majority of WSD participant recruitment was conducted via general practice lists, whereas previously local assessments had been conducted by local staff who visited patient’s homes, such as occupational therapists. According to one interviewee,

"(We were) all about how you use telehealth and telecare to improve health and social care for your population, which had nothing to do with randomised control trials. It caused massive damage in terms of what we would have been doing, because we had to stop doing what was obvious, which was helping those people that benefit most, and alter the direction of travel… Encouraging social services to refer their clients that could benefit from this was destroyed by the randomised control trial… So, it was very destructive in a sense (senior manager)."

Managers expressed the view that the sites needed to deviate from previous ‘real-life’ models of assessment and service delivery and create new trial-specific ones. During this process, some existing relationships and partnerships between stakeholders were damaged, and organisational goodwill was lost. As reported in other work [8] it was difficult for social and health care staff to morally accept that due to the randomisation requirements of the RCT, some people assessed as needing a service had to wait before the equipment was supplied:

"We’re very restricted in relation to WSD and, in fact, some of the people we referred to be included as part of WSD never got the kit … which leaves a bad taste because they did fit the criteria (social care professional). "

Previous telehealth and telecare services in Kent and Newham were led by local authority social services. To try and maximise previous learning both sites decided to keep this model. Partly with the aid of previous funding [34] telecare has already been widely implemented across the UK, and in two of the three demonstrator sites. Previous telecare experience assisted with recruitment but the fact that the sites could not use existing users in the RCT reduced the recruitment pool, leading to delays.

"We had already stripped out of Newham all of those people who could most benefit from telecare so they are outside of the trial… Anybody that really thought about where to actually do this would not choose somebody that had already done telecare (middle manager)."

Another challenge was the perceived uneven focus of the trial design, focusing separately on telecare and telehealth, with the latter seen as the most innovative part of the WSD trial. There was a strong sense that the RCT was more focused on telehealth rather than telecare interventions.

"Telehealth had a far greater exposure and it’s definite that the focus of the WSD was on the health service. Social care was always seen as an aside… It was very, very health focused, looking at mainly clinical and medical outcomes. So, no, I think it was really biased towards the NHS (social care professional)."

This emphasis on telehealth was hard for sites where social care staff led the programme, due to new reliance on the involvement of general practitioners (GPs). This problem was partly overcome in Kent when GP champions were placed within leading operational roles. These clinicians had the power, influence and knowledge to engage nursing teams and other GPs, they understood the extra work involved and the need for randomisation, and unlike many social care staff were not resistant to the constraints the trial imposed. Cornwall had less prior experience of remote care than the other two sites, so came into WSD with a relatively little telehealth and telecare infrastructure. The relative lack of an existing remote care system allowed them to use clinicians from the NHS Primary Care Trust (PCT) to lead the programme. The process of staff engagement was easier in Cornwall due to this decision. The PCT saw participation in WSD as an exciting opportunity to develop new system-wide care models, providing a springboard for their agenda for improved chronic disease management. As the trial progressed, it became clear that clinical champions, placed at strategic levels, helped sustain senior management interest, deemed essential for moving forward.

"I think having clinical champions as well as senior clinical managers out there saying ‘I have had really good success with this’ - I wouldn't want to lose that…When you hear that positive feedback, and we've made sure that’s all fed through to the PCT Board, then it’s hard for the PCT to say no, we’re not doing this (healthcare professional)."

### Organisational learning

For telehealth and telecare to stimulate ‘whole system redesign’ and integration, organisational flexibility and incremental, iterative learning was deemed essential by local management teams. However, achieving this flexibility was hindered by the requirements of the RCT. Many new processes, such as patient assessment and training, were set up according to the needs of the RCT protocol, but as large scale implementation progressed, it became clear that these processes were poorly aligned with the specific needs of the existing local context, but could not be adapted. According to one interviewee,

"There was certainly a period where we were rather locked into WSD being a discrete add-on service, as opposed to being an integrated whole system approach. That was influenced quite strongly by the need to not distort the evaluation process. So, we carried on doing some things that we knew were either expensive or just under par (middle manager)."

Unfortunately, as the programme progressed, the requirements of the RCT meant that many processes identified during implementation as locally sub-optimal, could not be significantly deviated from. As one manager put it,

"I think it has … made me realise about the limitations of RCTs. That whilst they are the gold standard in evidence, to some extent they don’t allow flexibility in terms of what you’d offer. Some of these sites have been working for two or three years, and I think … if left alone… they would be offering something different now than they were when they started, but we’ve restricted them from doing that (policy advisor)."

Staff reported they were able to learn about mistakes in implementing remote care, but keeping within the RCT protocol often meant that they could not take remedial action to rectify them. Not being able to put learning into practice was seen as detrimental to patient care. This affected staff morale and the introduction of change management strategies to support future large scale implementation and sustainability, after programme completion. In order to fit with local plans for scaling-up remote care, many of the processes developed for the WSD are now being gradually replaced, with the sites either developing new care models or reverting back to old ones.

"You have to unpick all the processes and procedures we put in place to deliver the RCT because they’re not good business processes. They’re too constrained so we’ve had to take everybody in that mind-set out of the programme environment (senior manager)."

Despite the impact of the RCT on the sites’ ambitions for mainstream implementation of remote care, its robust nature was nevertheless seen as extremely timely and pivotal to the future development of remote care services, both in the UK and worldwide. Most participants perceived ‘useful’ evidence as being partly subjective and context-specific, but they understood that this was not enough to ‘sell’ telehealth and ensure a long-term, widespread commitment to adoption. As reported in other remote care work [5] the need to gather systematically collected before-and-after data was seen as a top priority, with clinician engagement being greatly enhanced by the perceived robustness of the RCT.

### Moving forward

High levels of WSD staff turnover, and current uncertain economic conditions have all impacted on the sites’ future scalability plans. Time-limited funding meant many staff were employed specifically for the trial, but local spending plans meant that they had little opportunity to stay in post once the RCT recruitment was completed. In Newham this has been especially problematic as nearly all staff were employed as temporary ‘change management’ consultants. At the time of this paper all but two of these had left the organisation. According to one interviewee ‘*they’ve lost all that information and experience that they could have used and applied and driven it forward’ (middle manager).* More broadly, managers felt there was an over-emphasis on meeting RCT recruitment aims and research goals, at the expense of trying to understand the implementation levers and incentives that should be put in place to ensure that remote care made local business sense and could be further scaled-up and sustained. At the end of the RCT the sites were left starting from scratch in terms of building in and aligning implementation with local needs.

"The problem with the WSD programme was that … it required us by virtue of the numbers game to install telehealth in all sorts of people's homes. I think we now need to focus its use in the right places. Now that will mean taking some of the triallists off, and it will mean adding new patients on, where we are in control of the use of telehealth, rather than it being driven by the needs of the evaluation process (senior manager)."

Despite these setbacks, taking part in the programme has provided the sites with new infrastructure, equipment and resources. One of the programme achievements is that it has raised clinical awareness and trust in telehealth services. Many staff, originally sceptical about the efficacy of telehealth services, became enthused and excited by the innovation as their knowledge and interaction with the programme increased.

"I am one hundred and fifty percent committed and believe that telehealth is the way forward and I will be looking to work in a way that I can drive it forward and make it happen… So, absolutely it has changed me (healthcare professional)."

Less positively, this increased enthusiasm may provide a future source of tension if services developed in the programme are not scaled-up or sustained once the funding is removed. Concerns were raised that the telehealth services provided under WSD put increased demand on the local healthcare system, by serving to highlight previously hidden unmet social and clinical needs. The cost of meeting these unmet needs, in terms of additional resources, was seen as a serious barrier to scaling up.

"But I think we've also learnt that quite a component of telehealth has been picking up previously unmet need. My guess is that a lot of the activity on WSD has … made it better for people if they've been in touch with health services. But quite honestly, some of that need would not have been being met before (senior manager)."

## Discussion

The Whole System Demonstrator was specifically designed to provide more robust evidence of the effectiveness and cost-effectiveness of telehealth and telecare in the management of patients with long-term health conditions. In doing so, it was seen as a way of stimulating faster implementation of remote care. The RCT element of the evaluation means that questions about benefits evidence should in part be answered. However, the other aim of the programme, with telehealth and telecare acting as a driver for new levels of integration, and providing lessons for wide-scale future implementation, was less successful.

The original WSD evaluation design attempted to combine the need for better evidence, using an RCT, with lessons about organisational change and strategic decision making, to better inform decisions about scaling up and embedding the technology into mainstream services. The emerging results support the recent suggestion for less of a focus on conducting RCTs in health, that the quest for improved evidence does not necessarily align with an RCT approach [5,6,8], with more research needed which looks at ‘wicked’ [35] problems of the implementation and appropriateness of telecare/telehealth.

The components and constituent parts of the RCT did not exist in isolation from local implementation needs, and as the RCT was given priority these needs often became eroded or skewed. As with any pragmatic evaluation of this size, the final proposed design meant a compromise between methodological rigour and realism. A definitive RCT was required to produce the robust data policy makers and commissioners have been calling for. In contrast to other research designs that focus on knowledge in action [19], in order to produce robust, reliable and universal evidence, the RCT had to be largely implemented as originally planned and detached from the complexities of the environment in which the organisations were embedded. We know that organisational implementation and working practices are influenced by a wide range of complex local, political and individual processes [5,6,8]. Wide-scale rollout was not simply a question of increasing user numbers within a given domain and time span, and requires significant degrees of organisational learning about what works and what fails to work. Unfortunately, the artificiality of randomisation and associated levels of standardisation made a ‘learn, reflect and adopt’ approach very difficult. The RCT protocol was not well aligned with generating scalability lessons, iterative and participative modes of learning, and developing new levels of service integration. Action was often not possible until the trial was complete. Consequently, much of the iterative learning from the implementation process was not implemented.

Remote care technologies have often been positioned as a ‘cure all’, in both helping address the western world’s demographic time-bomb by filling gaps within the care system, and as a vehicle for new levels of system integration [36,37]. The White Paper from which the WSD programme originated states that it should ‘highlight the many barriers to realising this vision.’ [1] The idea that remote care implementation could lead to new levels of whole system working was always ambitious, with how this aim would be achieved was left ambiguous. As others have noted [38], while policy support may increase an organisation’s capacity to adopt an innovation, it will not change its strategic and cultural predisposition. This WSD stands out in being the largest trial of its type ever undertaken. Despite the resources deployed, and a comprehensive and rigorous site selection process of cases with past experience of telecare and telehealth, we found that in actuality the sites were not ready to implement this level of organisational change within the timeframe given. It appears that despite a rigorous site selection process, remote care was not a sufficiently powerful driver to significantly reduce fragmentation and discontinuities across the system, and push system integration significantly forward. Nor did engaging in the WSD programme create new levels of organisational readiness as originally envisioned. Even in Cornwall, where they started off with more flexibility, in that they had less devolved and of remote care working, telecare and telehealth did not lead to greater system integration. People from different parts of the health and social care system may have worked together more than previously, but the underlying structure of the systems in place remained largely unchanged [12]. What this study shows is that telehealth and telecare was successfully implemented locally without significant levels of system integration occurring and without the perceived need for this to happen. If work from the programme is to be locally sustained, perhaps the original goals of integration need to be redrawn in favour of more organic notions of incremental scaling-up, that pay attention to the evolving needs of the service-in-context as it grows [19]. While it seems unlikely that care system redesign on any significant scale could be implemented within the relatively short two year time-frame of the WSD, like previous research [6,12], these results suggest that even without the constraints imposed by the RCT, the ‘holy grail’ of health and social care integration driven by remote care, is still a very long way off. What is clear is that during the WSD programme, the RCT protocol led to implementation models that were not sufficiently sensitised to local contextual differences. An important lesson is that the development of a remote care service cannot occur in a contextual vacuum treated as a pre-wrapped generic implementation package to be adapted later. Locally sensitive levers and incentives must be factored in and co-designed both from inception and along the way.

## Conclusions

Our results suggest that more resources to support implementation and more evidence are not the whole answer to scaling-up remote care. Gathering evidence from large-scale RCTs, in parallel with implementation of remote care services, creates confusion. It becomes difficult to determine how much change is a product of trial processes, or directly attributable to changes in services delivery, and whether any of the observed changes will be sustainable [5,6,16]. In further investigating how remote care can be mainstreamed, away from the constraints of an RCT, the Department of Health has commissioned us to conduct parallel organisational research across six additional UK sites with varying levels of national and local government support, as well as research on the technology supply side. We are currently evaluating the findings. Early results suggest implementing telehealth and telecare systems more incrementally, at a pace that makes sense to the organisations of care in relation to their locally changing needs and priorities, is the best route to success.

## Competing interests

All the authors declare that they have no competing interests.

## Authors’ contributions

JH, (Planning and reporting the work) JB, (Guarantor) (Planning and reporting the work) TC, (Conducting, and reporting the work) The other authors: MK, AR, RF, JD, CS, PB, RB, and SN – all helped with planning and reporting the work. All authors read and approved the final manuscript.

## Funding

The study was funded by the Department of Health. The views expressed are those of the authors and are not necessarily those of the Department of Health.

## Pre-publication history

The pre-publication history for this paper can be accessed here:

http://www.biomedcentral.com/1472-6963/12/403/prepub
